# Correlation of mucosal healing endpoints with long-term clinical and patient-reported outcomes in ulcerative colitis

**DOI:** 10.1007/s00535-023-02013-7

**Published:** 2023-07-25

**Authors:** Gareth Parkes, Ryan C. Ungaro, Silvio Danese, Maria T. Abreu, Ethan Arenson, Wen Zhou, Dapo Ilo, F. Stephen Laroux, Huiwen Deng, Yuri Sanchez Gonzalez, Laurent Peyrin-Biroulet

**Affiliations:** 1 Dept of Gastroenterology, Royal London Hospital, Barts Health NHS Trust, London, UK; 2 Division of Gastroenterology, Icahn School of Medicine at Mount Sinai, New York, NY USA; 3 Gastroenterology and Endoscopy, IRCCS Ospedale San Raffaele and University Vita-Salute San Raffaele, Milan, Italy; 4 Division of Gastroenterology, Crohn’s and Colitis Center, University of Miami Miller School of Medicine, Miami, FL USA; 5 Adelphi Values, Boston, MA USA; 6 AbbVie Inc., Chicago, IL USA; 7 AbbVie Bioresearch Center, Worcester, MA USA; 8 Department of Pharmacy Systems Outcomes and Policy, University of Illinois at Chicago, Chicago, IL USA; 9 University Hospital of Nancy, Lorraine University, Vandoeuvre, France

**Keywords:** Histologic endoscopic mucosal improvement, Histologic endoscopic mucosal remission, Inflammatory bowel disease

## Abstract

**Background:**

We evaluated the clinical relevance of achieving histologic endoscopic mucosal improvement (HEMI) and the more stringent target of histologic endoscopic mucosal remission (HEMR) in the phase 3 maintenance trial of upadacitinib for moderately to severely active ulcerative colitis.

**Methods:**

Clinical and patient-reported outcomes were assessed in patients with clinical response after 8- or 16-week upadacitinib induction who received 52-week upadacitinib maintenance treatment. Cross-sectional and predictive analyses evaluated the relationship between HEMR or HEMI at Week 8/16 and Week 52, respectively, and outcomes at Week 52. Adjusted odds ratios (aOR) were derived from logistic regressions for patients achieving HEMR or HEMI without HEMR versus those not achieving HEMI.

**Results:**

Cross-sectional analyses showed that patients with HEMR had greater odds of achieving all clinical and patient-reported outcomes at Week 52 than those not achieving HEMI. In predictive analyses, patients with HEMR at Week 8/16 had significantly greater odds of achieving clinical remission (aOR = 3.6, *p* = 0.001) and endoscopic remission (aOR = 3.9, *p* < 0.001) at Week 52 than patients not achieving HEMI and HEMR. For patients achieving HEMI without HEMR, these odds were lower: clinical remission (aOR = 3.2, *p* < 0.001) and endoscopic remission (aOR = 2.4, *p* = 0.010). The odds of achieving clinically meaningful improvements in most patient-reported outcomes were directionally similar between HEMI and HEMR, but not statistically different to patients not achieving HEMI. No hospitalizations or surgeries were observed in patients with HEMR at Week 52.

**Conclusions:**

Achievement of HEMR or HEMI is clinically relevant with HEMR being associated with greater likelihood of improvement in long-term clinical and patient-reported outcomes. https://www.clinicaltrials.gov NCT02819635.

**Supplementary Information:**

The online version contains supplementary material available at 10.1007/s00535-023-02013-7.

## Introduction

Treatment of ulcerative colitis (UC), a chronic, progressive, debilitating inflammatory disease of the gastrointestinal tract [1] has evolved over time. Today, disease activity and therapeutic benefits can be evaluated by clinical symptoms, endoscopy, patient-reported outcomes, biomarkers, and histologic methods. The Selecting Therapeutic Targets in Inflammatory Bowel Disease (STRIDE-II) consensus guidelines recommend a treat-to-target approach in UC [2] to improve outcomes and reduce the risk of end-organ damage. Per STRIDE-II recommendations, short-term treatment targets include symptomatic relief along with normalization of serum and fecal inflammatory markers, while long-term treatment targets include clinical remission, endoscopic mucosal healing, and restoration of quality of life, as well as decreased rates of flares, dysplasia, and colectomy [2]. Histologic remission was suggested as an adjunct to endoscopic remission to achieve a deeper level of healing [2]. The STRIDE authors felt that there was insufficient data to have histologic healing as a treatment goal because few clinical studies report on the use of histologic endpoints and in those that do, the histologic endpoints have been achieved in a small proportion of patients.

Achievement of endoscopic healing has emerged as an important treatment goal for patients with UC as several studies suggest that endoscopic healing is associated with long-term clinical remission, decreased colectomy rates, and decreased rates of dysplasia [3–8]. Multiple scoring systems are available to assess endoscopic activity, which has resulted in variable definitions of endoscopic remission in clinical trials [9]. The Mayo endoscopic score (MES) [10] and Ulcerative Colitis Endoscopic Index of Severity (UCEIS) [11] are commonly used to assess endoscopic healing, although the exact definition of endoscopic healing remains to be established [9]. Published studies suggest that more stringent endoscopic criteria may provide even better long-term benefits for patients [12–15]. Barreiro-de Acosta et al. [12] showed that patients who achieved MES = 0 (inactive disease) had a lower relapse rate at 12 months than those who achieved MES = 1 (mild disease). A recent meta-analysis [13] demonstrated that patients with MES = 0 had a 52% lower risk of clinical relapse at 12 months than patients with MES = 1. Persistent histologic inflammation is often observed in patients who achieve endoscopic healing [16–19] and is associated with an increased risk of relapse, colectomy, and colorectal neoplasia [8, 19–25]. More recently, histologic remission was shown to be associated with more favorable outcomes, including lower hospitalization and relapse rates, and lower cancer risk [14].

At present, there are few data available on the impact of a composite histologic and endoscopic endpoint on longer-term outcomes, particularly early in the treatment cycle. Two endpoints that combine endoscopic and histologic disease activity have been proposed and are currently being utilized in clinical trial settings [26–30]. The first composite endpoint is histologic endoscopic mucosal improvement (HEMI), defined as MES ≤ 1 and Geboes histologic score ≤ 3.1 [27, 28]. The second endpoint is a more stringent, novel composite endpoint, and histologic endoscopic mucosal remission (HEMR) or deep mucosal healing, defined as MES = 0 and Geboes histologic score < 2.0. We therefore aimed to investigate the effect of achieving a composite of endoscopic remission and histologic remission in UC early in the treatment course on long-term clinical outcomes in patients treated with upadacitinib in the U-ACHIEVE maintenance trial.

## Methods

### Study design and data source

The upadacitinib phase 3 program consists of 2 identical induction studies (U-ACHIEVE induction and U-ACCOMPLISH) and 1 maintenance study (U-ACHIEVE maintenance). In the induction studies, patients were randomized to receive upadacitinib 45 mg or placebo for 8 weeks. At Week 8, patients who did not achieve a clinical response were eligible to receive an additional 8 weeks of treatment with upadacitinib 45 mg. Patients who achieved a clinical response following upadacitinib 45 mg for 8 or 16 weeks were eligible to enroll in the U-ACHIEVE maintenance study and were randomized 1:1:1 to receive upadacitinib 15 mg, upadacitinib 30 mg, or placebo. Randomization was stratified by bio-IR status (bio-IR vs non-bio-IR), corticosteroid use (yes or no), and Adapted Mayo score (≤ 7 or > 7) at Baseline. Within bio-IR, the randomization was further stratified by number of prior biologic treatments (≤ 1 or > 1). Within non-bio-IR, the randomization was further stratified by previous biologic use (yes or no). We performed post hoc analyses of data from the Phase 3 U-ACHIEVE upadacitinib maintenance trial to evaluate the relationship between the HEMR/HEMI endpoints and achievement of long-term clinical and patient-reported outcomes at Week 52. Histologic, clinical, and patient-reported outcomes data from the Phase 3 U-ACHIEVE [NCT02819635] upadacitinib maintenance trial were evaluated in this analysis. The objective of the U-ACHIEVE maintenance trial was to evaluate the efficacy and safety of upadacitinib compared with placebo in achieving clinical remission in patients with moderately to severely active UC who had a clinical response per adapted Mayo score following induction with upadacitinib 45 mg once daily (QD). In the maintenance trial, patients were randomized 1:1:1 to upadacitinib 15 mg QD, upadacitinib 30 mg QD, or placebo QD. Details of the U-ACHIEVE induction and maintenance trials are reported elsewhere [28].

A full colonoscopy was performed for all patients at screening. At Weeks 8 and 52, endoscopies were either a colonoscopy or a flexible sigmoidoscopy, depending on the extent of disease at screening, and were performed up to the segment where a clear demarcation of inflammation was observed. In patients who required 16 weeks of upadacitinib induction therapy, an additional colonoscopy or a flexible sigmoidoscopy was performed at Week 16. During all endoscopies, biopsies for histologic evaluation were taken from the rectosigmoid colon (approximately 15–30 cm from the anal verge) and from the area of most inflammation. For follow-up endoscopies, in the absence of any visible lesions or areas of general inflammation characteristic of UC, biopsies were to be collected from normal mucosa in the same segments as noted above. Tissue samples were processed, mounted to slides, and digitized using a whole slides scanner. For each image, a central reader performed the reading and provided a histologic score. Both histologic and endoscopic scoring were performed by the central readers, who were properly trained regarding lesion definition and identification, proficient in scoring and blinded to other clinical or study data. The pathologists who participated as blinded central readers have extensive experience as pathologists (14, 17, 20, and 28 years) and specifically in the field of gastrointestinal pathology (8, 9, 12, and 16 years, respectively).

The Geboes histologic score is an index commonly used to measure histologic disease activity in UC, which has undergone content and construct validation and reliability testing [31–34]. Patients are assigned a Geboes histologic score between 0 and 5.4, based on the results from an endoscopy with biopsy. Higher scores indicate greater levels of inflammation, with the scores used to distinguish between inactive disease (grade 0 or 1), mildly active disease (grade 2 or 3), or moderate to severely active disease (grade 4 or 5).

The U-ACHIEVE maintenance trial was conducted in accordance with the protocol, International Conference on Harmonization (ICH) guidelines, applicable regulations and guidelines governing clinical study conduct and the ethical principles that have their origin in the Declaration of Helsinki. All participants provided written informed consent before any study-related procedures were performed. All authors had access to the study data and reviewed and approved the final manuscript.

### Composite histologic endoscopic endpoints

#### HEMI

HEMI is a composite endpoint defined as a Mayo endoscopic sub-score of 0 or 1 and a Geboes histologic score ≤ 3.1 [27, 28]. Achieving the HEMI endpoint indicates improvement in the macroscopic appearance of the mucosal surface, as well as improvement in the microscopic and cellular features characteristic of mucosal inflammation. The Mayo endoscopic sub-score of 0 or 1 is defined by the lack of marked erythema, no friability, absence of vascular pattern and erosions, and no spontaneous bleeding or ulcerations that are frequently observed in patients with moderate to severe UC.

#### HEMR

HEMR is a novel, more stringent composite endpoint defined as a Mayo endoscopic sub-score of 0 and a Geboes histologic score < 2.0. Achieving the HEMR endpoint indicates that the mucosa appears normal upon endoscopic inspection and that there are no neutrophils in crypts or lamina propria, and no increase in eosinophils, no crypt destruction, and no erosions, ulcerations, or granulation tissue.

### Outcomes assessed at Week 52

Clinical outcomes included corticosteroid-free remission, sustained clinical response defined as a decrease in the Adapted Mayo score ≥ 2 points and ≥ 30% from baseline, plus a decrease in rectal bleeding score (RBS) ≥ 1 or an absolute RBS ≤ 1 at Week 8/16 that was maintained at Week 52, clinical remission per full and adapted Mayo score, endoscopic improvement and remission (RBS = 0, SFS ≤ 1), and fecal calprotectin (FCP) levels ≤ 250 μg/g and ≤ 150 μg/g (Table 1). In addition to RBS and SFS, other patient-reported outcome measures included the Functional Assessment of Chronic Illness Therapy–Fatigue (FACIT-F) questionnaire, Ulcerative Colitis Symptoms Questionnaire (UC-SQ), Inflammatory Bowel Disease Questionnaire (IBDQ), Short Form Health Survey (SF-36) physical component summary (PCS) and mental component summary (MCS) scores, European Quality of Life Five Dimensions Five Levels (EQ-5D-5L) index, and Work Productivity and Activity Impairment (WPAI) questionnaire.

**Table 1:** Baseline characteristics of patients in U-ACHIEVE maintenance trial (intent-to-treat)

Characteristic	Placebo (*n* = 149)	Upadacitinib 15 mg OD (*n* = 148)	Upadacitinib 30 mg OD (*n* = 154)
Female, *n* (%)	64 (43.0)	53 (35.8)	68 (44.2)
Race, *n* (%)			
Caucasian	93 (62.4)	97 (65.5)	101 (65.6)
Black or African American	6 (4.0)	7 (4.7)	3 (1.9)
Asian	42 (28.2)	44 (29.7)	48 (31.2)
American Indian or Alaska Native	0	0	0
Native Hawaiian or other Pacific Islander	1 (0.7)	0	1 (0.6)
Multiple	7 (4.7)	0	1 (0.6)
Age (years), median (IQR)	40.0 (21.0)	40.0 (22.0)	41.0 (7.0)
Disease duration (years), median (IQR)	6.2 (8.6)	6.4 (10.6)	6.0 (9.7)
Previous failure with biologic therapy, *n* (%)	81 (54.4)	71 (48.0)	73 (47.4)
Endoscopic sub-score			
= 3, *n* (%)	98 (65.8)	100 (65.6)	108 (70.1)
Mean ± SD	2.7 ± 0.48	2.7 ± 0.47	2.7 ± 0.48

Baseline demographics and disease characteristics were measured at baseline during the induction studies

*IQR* interquartile range, *OD* once daily, *SD* standard deviation

### Data analysis

Data from patients who achieved a clinical response after 8 or 16 weeks of upadacitinib induction treatment and received upadacitinib maintenance treatment were analyzed in this study. Non-responder imputation was conducted in all Week 52 outcomes with no special data handling for missing data because of corona virus disease of 2019 (COVID-19). Missing data were not imputed for HEMI or HEMR at Week 8/16.

To assess the relative importance of HEMR compared with HEMI, cross-sectional and predictive analyses were conducted on the following patient groups: (1) patients who achieved HEMR, (2) patients who achieved HEMI without HEMR, and (3) patients who did not achieve HEMI. The cross-sectional analysis examined the likelihood of achieving long-term clinical and patient-reported outcomes at Week 52 among patients who achieved HEMR or HEMI without HEMR versus those who did not achieve HEMI. Sensitivity analyses compared outcomes in patients with vs without HEMR and with vs without HEMI. Predictive analysis assessed the relationship between achieving HEMR or HEMI without HEMR at the end of induction (Week 8/16) and outcomes at Week 52.

Clinically meaningful improvement in patient-reported outcomes was assessed as the likelihood of achieving a change from baseline (before starting treatment) in the patient-reported outcome score ≥ the corresponding meaningful within-patient change threshold (MWPC). The MWPC thresholds for UC-SQ (≥ 10) and FACIT-F (≥ 5) were estimated from anchor- and distribution-based analyses of upadacitinib phase 2 data. MWPC thresholds for IBDQ (≥ 16), [35–37] SF-36 PCS and MCS (≥ 4.1), [38] EQ-5D-5L index (≥ 0.076), [39] and WPAI (work time missed ≥ 6.5, impairment while working ≥ 6.1, overall work impairment ≥ 7.3, activity impairment ≥ 8.5) [40] were extracted from published literature.

Odds ratios (ORs) with 95% confidence intervals (CIs) were derived from logistic regression adjusting for the following characteristics: histologic score at baseline (continuous), maintenance treatment dosage (high or low), gender (male or female), disease extent (left-sided or extensive), disease duration, and baseline age and weight.

The percentages of patients who achieved HEMR or HEMI without HEMR at end of induction (Week 8/16) and attained long-term clinical and patient-reported outcomes at Week 52 were determined. The percentages of patients achieving these histological endpoints at end of maintenance (Week 52) and clinical and patient-reported outcomes at Week 52 were also calculated. Chi-square test was used to determine the significance test for both mucosal healing endpoints (HEMR and HEMI without HEMR) compared with no HEMI.

To further assess the benefits of the HEMI/HEMR end points, the number of UC-related hospitalizations and surgeries was tabulated and stratified by mutually exclusive HEMI and HEMR categories at Week 8/16 (end of induction) and Week 52 (end of maintenance). However, it was not feasible to perform logistic regression analyses because of the limited number of hospitalizations and surgeries observed in the upadacitinib trial data.

## Results

### Demographic data showed that nearly half of the patients were previously treated with biologics

Median age and median duration of disease of the study population who participated in the maintenance trial were approximately 40 years and 6 years, respectively (Table 1). At least 47% of the study population previously failed biologic therapy and at least 65% had an endoscopic sub-score of 3 at baseline of induction study. Of 125 patients who had a clinical response at end of induction (Week 16), 41.6% lost their clinical response at Week 52 (end of maintenance).

### Patients who achieved HEMR had greater likelihood of improvement in clinical and patient-reported outcomes than those who achieved HEMI alone

**Fig. 1:**
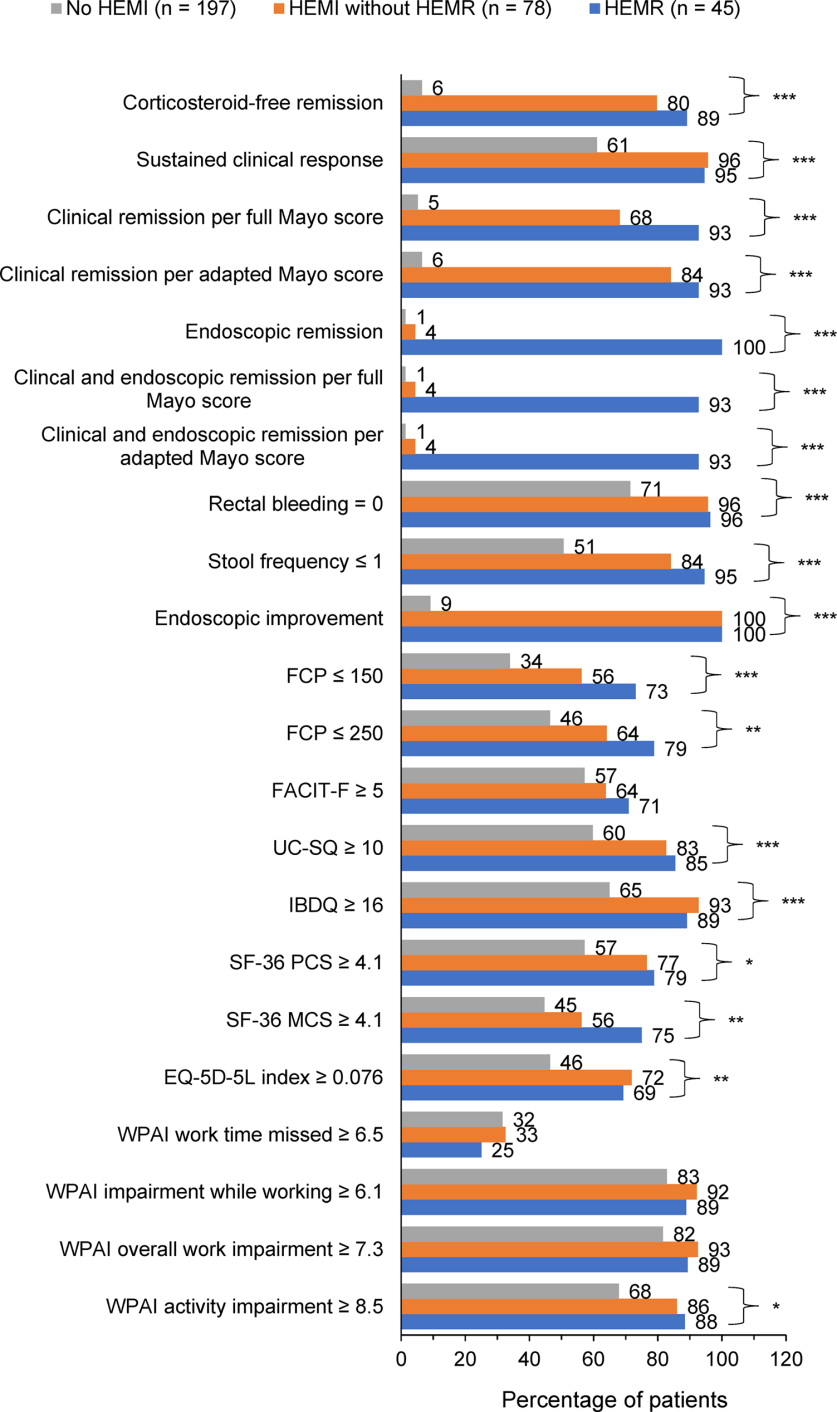
Cross-sectional analysis: Percentage of patients who achieved HEMR or HEMI without HEMR at Week 52 (end of maintenance) and outcomes at Week 52. *p < 0.05, **p < 0.01, ***p ≤ 0.001 for joint significance of (HEMR and HEMI without HEMR) versus no HEMI. *EQ-5D-5L* European Quality of Life Five Dimensions Five Levels, *FACIT-F* Functional Assessment of Chronic Illness Therapy–Fatigue, *FCP* fecal calprotectin, *HEMI* histologic endoscopic mucosal improvement, *HEMR* histologic endoscopic mucosal remission, *IBDQ* Inflammatory Bowel Disease Questionnaire, *SF-36 MCS* Short Form Health Survey Mental Component Summary, *SF-36 PCS* Short Form Health Survey Physical Component Summary, *UC-SQ* Ulcerative Colitis Symptoms Questionnaire

For the cross-sectional analysis, an equal or greater percentage of patients who achieved HEMR at Week 52 attained improved clinical outcomes and had clinically meaningful improvements from baseline in patient-reported outcomes at Week 52 compared with patients who achieved HEMI alone (Fig. 1). The cross-sectional regression analysis showed that patients who achieved HEMR (versus no HEMI) at Week 52 had significantly greater odds of attaining all clinical outcomes, including corticosteroid-free remission, sustained clinical response, and clinical and endoscopic remission per full Mayo and per adapted Mayo score at Week 52 (Table 2). These patients also had significantly greater odds of achieving clinically meaningful improvements in UC-SQ, IBDQ, SF-36 PCS, SF-36 MCS, EQ-5D-5L, and WPAI activity impairment at Week 52 (Table 2). Furthermore, the odds of improvement in all clinical outcomes and in FACIT-F, UC-SQ, SF-36 PCS, SF-36 MCS, and overall work impairment were numerically higher in patients who achieved HEMR vs no HEMI compared with those who achieved HEMI alone vs no HEMI.

**Table 2:** Cross-sectional analyses: Adjusted odds ratios for HEMR or HEMI without HEMR at Week 52 (end of maintenance) and clinical outcomes at Week 52 in U-ACHIEVE

Outcomes at Week 52^a^	Single regression per outcome for HEMR and HEMI without HEMR vs no HEMI			
	HEMR (*n* = 55^b^) vs no HEMI (*n* = 77^b^)		HEMI (*n* = 69^b^) without HEMR vs no HEMI (*n* = 77^b^)	
	Adjusted odds ratio (95% CI)^c^	*p* value	Adjusted odds ratio (95% CI)^c^	*p* value
Clinical outcomes				
Corticosteroid-free remission^b,d^	476.3 (71.4, > 999.99)	< .001	119.2 (23.9, 594.9)	< .001
Sustained clinical response^b,e^	25.0 (5.4, 115.5)	< .001	13.5 (3.3, 54.8)	< .001
Clinical remission per full Mayo score^f^	> 999.99 (180.2, > 999.99)	< .001	69.2 (16.1, 296.9)	< .001
Clinical remission per adapted Mayo score^b,g^	> 999.99 (157.0, > 999.99)	< .001	253.4 (41.6, > 999.99)	< .001
Endoscopic remission^b,h^	N/A due to HEMR definition		13.0 (0.6, 294.2)	.106
Clinical and endoscopic remission per full Mayo score^b,f,h^	> 999.99 (210.5, > 999.99)	< .001	5.2 (0.41, 65.4)	.204
Clinical and endoscopic remission per adapted Mayo score^b,g,h^	> 999.99 (210.5, > 999.99)	< .001	5.2 (0.41, 65.4)	.204
Rectal bleeding = 0^b^	22.5 (4.0, 126.9)	< .001	7.3 (1.8, 29.2)	.005
Stool frequency ≤ 1^b^	34.2 (7.9, 147.4)	< .001	4.9 (2.0, 12.2)	< .001
Endoscopic improvement^b,i^	*N*/*A* due to HEMI and HEMR definitions			
FCP ranges^j^				
FCP ≤ 150 μg/g	6.9 (2.7, 17.3)	< .001	3.7 (1.6, 8.6)	.003
FCP ≤ 250 μg/g	6.1 (2.4, 15.7)	< .001	2.8 (1.2, 6.4)	.015
Clinically meaningful improvement from induction baseline in patient-reported outcomes^k^				
FACIT-F (≥ 5)^b^	2.0 (0.9, 4.4)	.100	1.5 (0.7, 3.1)	.316
UC-SQ (≥ 10)^b^	4.5 (1.7, 11.9)	.002	2.9 (1.2, 6.9)	.016
IBDQ (≥ 16)^b^	4.2 (1.5, 11.7)	.007	7.0 (2.2, 22.6)	.001
SF-36 PCS (≥ 4.1)^j^	4.1 (1.6, 10.4)	.004	2.9 (1.2, 7.0)	.018
SF-36 MCS (≥ 4.1)^j^	4.9 (2.0, 12.2)	< .001	1.7 (0.8, 3.9)	.186
EQ-5D-5L index (≥ 0.076)^j^	4.5 (1.8, 11.1)	.001	4.8 (1.9, 12.0)	< .001
WPAI				
Work time missed (≥ 6.5)^l^	0.9 (0.3, 2.8)	.817	1.3 (0.4, 3.8)	.672
Impairment while working (≥ 6.1)^l^	2.7 (0.4, 16.5)	.281	3.0 (0.6, 15.0)	.191
Overall work impairment (≥ 7.3)^l^	3.4 (0.6, 19.6)	.173	2.9 (0.6, 14.0)	.197
Activity impairment (≥ 8.5)^j^	3.7 (1.3, 10.8)	.016	4.5 (1.6, 12.6)	.005

*CI* confidence interval, *EQ-5D-5L* European Quality of Life Five Dimensions Five Levels, *FACIT-F* Functional Assessment of Chronic Illness Therapy–Fatigue, *FCP* fecal calprotectin, *HEMI* histologic endoscopic mucosal improvement, *HEMR* histologic endoscopic mucosal remission, *IBDQ* Inflammatory Bowel Disease Questionnaire, *N/A* not applicable, *NRI-NC* non-responder imputation with no special data handling for missing due to COVID-19, *SF-36 MCS* Short Form Health Survey Mental Component Summary, *SF-36 PCS* Short Form Health Survey Physical Component Summary, *UC* ulcerative colitis, *UC-SQ* Ulcerative Colitis Symptoms Questionnaire, *WPAI* Work Productivity and Activity Impairment questionnaire

^a^NRI-NC was conducted in all Week 52 outcomes

^b^*n* = 201, includes patients who achieved a clinical response after 8 weeks or 16 weeks of upadacitinib induction treatment

^c^Adjusted for maintenance baseline Geboes histologic score, dosage, gender, age, weight, UC disease extent, UC disease duration, and use of extended therapy (16-week induction period). Due to the adjustment of covariates, patients with missing values on the covariates were dropped in the logistic regressions

^d^Corticosteroid-free remission was defined as achieving 90-day steroid-free clinical remission per adapted Mayo in patients who achieved clinical remission at the end of induction treatment

^e^Sustained clinical response was defined as remained clinically responsive at the end of Week 52

^f^Clinical remission per full Mayo was defined as total Mayo score ≤ 2 with no sub-score > 1

^g^Clinical remission per adapted Mayo (full Mayo excluding physician’s global assessment) was defined as stool frequency sub-score ≤ 1 and not greater than baseline, rectal bleeding sub-score = 0, endoscopic sub-score ≤ 1 without friability

^h^Endoscopic remission defined as Mayo endoscopic sub-score = 0

^i^Endoscopic improvement defined as Mayo endoscopic sub-score ≤ 1

^j^*n* = 172, includes only patients who achieved a clinical response after 8 weeks of upadacitinib induction treatment

^k^Clinically meaningful improvement in patient-reported outcomes was assessed as the likelihood of achieving a change from induction baseline in patient-reported outcome score ≥ the corresponding meaningful within-patient change threshold

^l^Only includes patients who had baseline WPAI scores. *n* = 154 for work time missed, *n* = 142 for impairment while working, *n* = 154 for overall work impairment

**Fig. 2:**
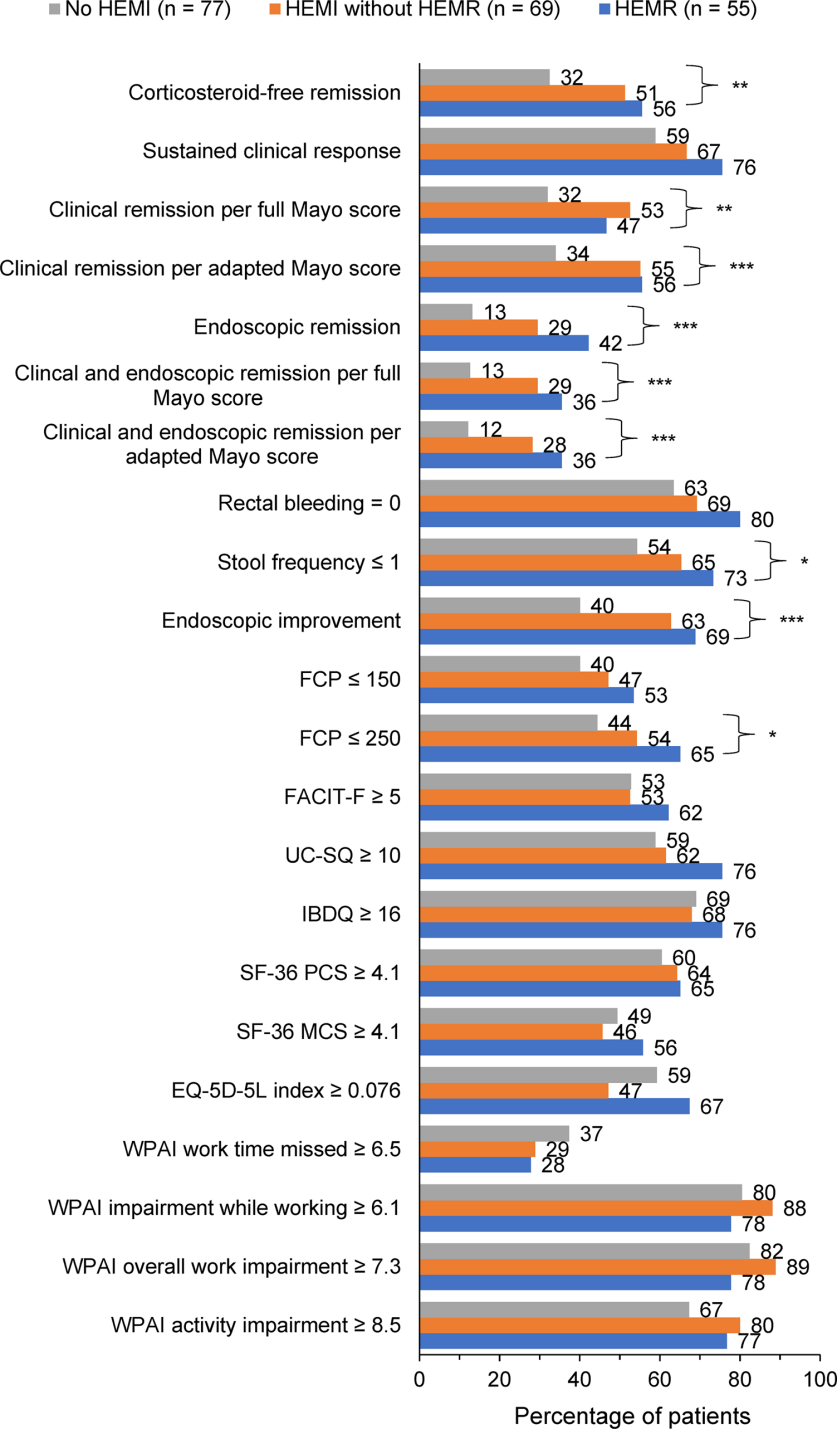
Predictive analysis: Percentage of patients who achieved HEMR or HEMI without HEMR at Week 8/16 (end of induction) and outcomes at Week 52 (end of maintenance). *p < 0.05, **p < 0.01, ***p ≤ 0.001 for joint significance of (HEMR and HEMI without HEMR) versus no HEMI. *EQ-5D-5L* European Quality of Life Five Dimensions Five Levels, *FACIT-F* Functional Assessment of Chronic Illness Therapy–Fatigue, *FCP* fecal calprotectin, *HEMI* histologic endoscopic mucosal improvement, *HEMR* histologic endoscopic mucosal remission, *IBDQ* Inflammatory Bowel Disease Questionnaire, *SF-36 MCS* Short Form Health Survey Mental Component Summary, *SF-36 PCS* Short Form Health Survey Physical Component Summary, *UC-SQ* Ulcerative Colitis Symptoms Questionnaire

An equal or greater percentage of patients with HEMR at Week 8/16 attained clinical outcomes and had clinically meaningful improvements from baseline (before starting treatment) in patient-reported outcomes at Week 52 compared to patients who achieved HEMI without HEMR in the predictive analysis (Fig. 2). The predictive regression analyses showed that achievement of HEMR (vs no HEMI) at Week 8/16 was associated with independently and significantly greater odds of attaining all clinical outcomes (Table 3). Achievement of HEMR (vs no HEMI) at Week 8/16 was associated with greater odds of attaining clinically meaningful improvements from induction baseline in patient-reported outcomes at Week 52, although the improvements were not statistically significant (Table 3). Similar results were observed for patients who achieved HEMI versus no HEMI. However, it should be noted that the odds of attaining most clinical outcomes and improvements in most patient-reported outcomes were numerically higher in patients who achieved HEMR vs no HEMI compared with those who achieved HEMI alone vs no HEMI.

**Table 3:** Predictive analyses: adjusted odds ratios for HEMR or HEMI without HEMR at Week 8/16 (end of induction) and clinical outcomes at Week 52 in U-ACHIEVE

Outcomes at Week 52^a^	Single regression per outcome for HEMR and HEMI without HEMR vs no HEMI			
	HEMR (*n* = 45^b^) vs no HEMI (*n* = 197^b^)		HEMI without HEMR (*n* = 78^b^) vs no HEMI (*n* = 197^b^)	
	Adjusted odds ratio (95% CI)^c^^i^	*P* value	Adjusted odds ratio (95% CI)^c^	*P* value
Clinical outcomes				
Corticosteroid-free remission^b,d^	3.8 (1.8, 8.2)	< 0.001	2.8 (1.6, 5.1)	< 0.001
Sustained clinical response^b,e^	2.8 (1.2, 6.5)	.014	1.9 (1.0, 3.4)	.044
Clinical remission per full Mayo score^b,f^	2.4 (1.2, 5.2)	.019	2.9 (1.6, 5.3)	< 0.001
Clinical remission per adapted Mayo score^b,g^	3.6 (1.7, 7.7)	.001	3.2 (1.7, 5.7)	< 0.001
Endoscopic remission^b,h^	3.9 (1.7, 8.6)	< 0.001	2.4 (1.2, 4.7)	.010
Clinical and endoscopic remission per full Mayo score^b,f,h^	3.2 (1.4, 7.2)	.006	2.7 (1.4, 5.3)	.004
Clinical and endoscopic remission per adapted Mayo score^b,g,h^	3.5 (1.5, 7.9)	.003	2.7 (1.3, 5.3)	.006
Rectal bleeding = 0^b^	3.5 (1.4, 8.6)	.006	1.8 (1.0, 3.3)	.067
Stool frequency ≤ 1^b^	2.7 (1.2, 6.0)	.015	2.0 (1.1, 3.6)	.022
Endoscopic improvement^b,i^	5.0 (2.2, 11.3)	< 0.001	3.3 (1.8, 5.9)	< 0.001
FCP ranges^j^				
FCP ≤ 150 μg/g	2.3 (1.1, 5.0)	.032	1.6 (0.9, 2.9)	.122
FCP ≤ 250 μg/g	3.3 (1.5, 7.3)	.004	1.9 (1.1, 3.6)	.032
Clinically meaningful improvement from induction baseline in patient-reported outcomes^k^				
FACIT-F (≥ 5)^b^	1.5 (0.7, 3.0)	.314	1.1 (0.6, 1.9)	.753
UC-SQ (≥ 10)^b^	2.7 (1.2, 6.0)	.019	1.4 (0.8, 2.4)	.296
IBDQ (≥ 16)^b^	1.3 (0.6, 3.0)	.483	1.1 (0.6, 1.9)	.863
SF-36 PCS (≥ 4.1)^j^	1.2 (0.6, 2.6)	.603	1.4 (0.8, 2.6)	.277
SF-36 MCS (≥ 4.1)^j^	1.2 (0.6, 2.6)	.560	0.9 (0.5, 1.7)	.837
EQ-5D-5L index (≥ 0.076)^j^	1.4 (0.6, 3.0)	.413	0.8 (0.4, 1.4)	.348
WPAI				
Work time missed (≥ 6.5)^l^	0.8 (0.2, 2.8)	.758	0.7 (0.3, 1.6)	.414
Impairment while working (≥ 6.1)^l^	2.0 (0.4, 10.1)	.408	2.7 (0.8, 9.0)	.115
Overall work impairment (≥ 7.3)^l^	1.9 (0.4, 9.6)	.420	2.1 (0.7, 6.5)	.204
Activity impairment (≥ 8.5)^j^	1.6 (0.7, 3.6)	.298	2.4 (1.2, 5.0)	.016

*CI* confidence interval, *EQ-5D-5L* European Quality of Life Five Dimensions Five Levels, *FACIT-F* Functional Assessment of Chronic Illness Therapy–Fatigue, *FCP* fecal calprotectin, *HEMI* histologic endoscopic mucosal improvement, *HEMR* histologic endoscopic mucosal remission, *IBDQ* Inflammatory Bowel Disease Questionnaire, *NRI-NC* non-responder imputation with no special data handling for missing due to COVID-19, *SF-36 MCS* Short Form Health Survey Mental Component Summary, *SF-36 PCS* Short Form Health Survey Physical Component Summary, *UC* ulcerative colitis, *UC-SQ* Ulcerative Colitis Symptoms Questionnaire, *WPAI* Work Productivity and Activity Impairment questionnaire

^a^NRI-NC was conducted in all Week 52 outcomes. No missing data imputation was used for HEMI and HEMR at Week 8/16

^b^*n* = 320, includes patients who achieved a clinical response after 8 weeks or 16 weeks of upadacitinib induction treatment

^c^Adjusted for maintenance baseline Geboes histologic score, dosage, gender, age, weight, UC disease extent, UC disease duration, and use of extended therapy (16-week induction period). Due to the adjustment of covariates, patients with missing values on the covariates were dropped in the logistic regressions

^d^Corticosteroid-free remission was defined as achieving 90 days steroid-free clinical remission per adapted Mayo in patients who achieved clinical remission at the end of induction treatment

^e^Sustained clinical response was defined as remained clinically responsive at the end of Week 52

^f^Clinical remission per full Mayo was defined as total Mayo score ≤ 2 with no sub-score > 1

^g^Clinical remission per adapted Mayo (full Mayo excluding physician’s global assessment) was defined as stool frequency sub-score ≤ 1 and not greater than baseline, rectal bleeding sub-score = 0, endoscopic sub-score ≤ 1 without friability

^h^Endoscopic remission defined as Mayo endoscopic sub-score = 0

^i^Endoscopic improvement defined as Mayo endoscopic sub-score ≤ 1

^j^*n* = 275, includes only patients who achieved a clinical response after 8 weeks of upadacitinib induction treatment

^k^Clinically meaningful improvement in patient-reported outcomes was assessed as the likelihood of achieving a change from induction baseline in patient-reported outcome score ≥ the corresponding meaningful within-patient change threshold

^l^Only includes patients who had baseline WPAI scores. *n* = 154 for work time missed, *n* = 142 for impairment while working, *n* = 154 for overall work impairment

### Patients who achieved HEMR had improved outcomes compared with those who did not achieve HEMR

The cross-sectional regression analysis (Supplemental Table 1) demonstrated that achieving HEMR (versus not achieving HEMR) at Week 52 significantly increased the odds of achieving all clinical outcomes, as well as the increasing the odds of achieving clinically meaningful improvements from induction baseline in UC-SQ, SF-36 PCS, and SF-36 MCS. The predictive regression analysis (Supplemental Table 2) showed that achieving HEMR (versus not achieving HEMR) at Week 8/16 significantly increased the odds of attaining most clinical outcomes at Week 52 and numerically increased the odds of achieving clinically meaningful improvements from induction baseline in patient-reported outcomes at Week 52.

### Patients who achieved HEMI had improved outcomes compared with those who did not achieve HEMI

The cross-sectional regression analysis demonstrated that achieving HEMI at Week 52 was associated with significantly greater odds of achieving all clinical outcomes at Week 52, as well as clinically meaningful improvements in UC-SQ, IBDQ, SF-36 PCS and MCS, EQ-5D-5L, and the WPAI activity impairment domain (Supplemental Table 1).

The predictive regression analysis showed that achieving HEMI (versus not achieving HEMI) at Week 8/16 was associated with significantly greater odds of achieving all clinical outcomes at Week 52 (Supplemental Table 2). The odds of achieving clinically meaningful improvements from induction baseline in patient-reported outcomes were increased, but not statistically significant for most outcomes.

### Correlation of Geboes score with long-term clinical outcomes

The relationship between the Geboes score and long-term clinical outcomes was also evaluated. Patients were assigned to the following Geboes score intervals [0, 2.0), [2.0, 3.1], and (3.1, 5.4] based on the histologic evaluation of biopsy samples taken during the U-ACHIEVE maintenance trial. These intervals were selected to assess patients who achieved the histology threshold for mucosal healing (Geboes score < 2.0) and those who achieved the histologic mucosal improvement threshold without mucosal healing [2.0, 3.1]. Patients with GS < 2.0 at Week 52 had significantly greater odds of achieving all clinical endpoints at Week 52 compared to patients with GS > 3.1 (Supplemental Fig. 1). Patients with GS < 2.0 at end of induction had a significantly higher likelihood of achieving clinical remission per adapted Mayo score (OR = 1.8, *P* < 0.030) and endoscopic improvement (OR = 1.8, *P* < 0.015) at Week 52 than patients with GS > 3.1. Results were directionally similar, but not statistically significant, for all other outcomes (Supplemental Fig. 2).

In addition, we calculated Spearman correlations for the Geboes score and secondary measures at baseline and Week 8 (end of induction). At Week 8, moderate (*r* ≥ 0.3) to strong (*r* ≥ 0.5) positive Spearman correlations were found between the Geboes score and Mayo subscores as well as the Mayo Full and Partial score (Supplemental Table 3).

### No hospitalizations or surgeries were observed during the 52-week maintenance phase in patients with HEMR at Week 8/16

None of the patients who achieved HEMR at Week 8/16 (*n* = 45) or Week 52 (*n* = 55) had an UC-related hospitalization or surgery during the 52-week maintenance phase (Supplemental Tables 4 and 5). In contrast, 4 patients who did not achieve HEMR (*n* = 275 by Week 8/16; *n* = 146 by Week 52) had an UC-related hospitalization or surgery during the 52-week maintenance phase. Of these 4 patients, 1 achieved HEMI only and the other 3 patients did not achieve HEMI.

## Discussion

In this post hoc analysis of patients with UC treated with upadacitinib, we observed that both HEMR and HEMI were associated with better long-term clinical outcomes. Achievement of HEMR at Week 8/16 was associated with numerically greater odds of improved clinical and patient-reported outcomes at Week 52 compared with patients who achieved HEMI without HEMR. Also, no UC-related hospitalizations and surgeries were observed during the 52-week maintenance phase in patients who achieved HEMR at Week 8/16.

Results obtained in the present analysis support the long-term benefits to patients who achieve the stringent endpoint of HEMI. The HEMI endpoint combines both endoscopic and histologic improvement cutoffs (MES of 0 or 1 and Geboes histologic score ≤ 3.1, respectively). Patients who achieve the HEMI endpoint gained improvement in the macroscopic appearance of the mucosal surface as well as improvement in the microscopic and cellular features that are characteristic of mucosal inflammation [14, 31, 41]. The endoscopic improvement (MES = 0 or 1) portion of the HEMI score is defined by lack of marked erythema, no friability, absence of vascular pattern and erosions, and no spontaneous bleeding or ulcerations [31, 41]. Patients who achieve low MES scores (0 or 1) have been shown to have a lower risk of relapse than those who do not. Achieving the histologic threshold of Geboes histologic score ≤ 3.1 indicates that < 5% of the mucosal crypts have neutrophilic infiltrate and there is an absence of crypt destruction (Grade 4) or ulceration (Grade 5) [31]. Post hoc analysis of data from the VARSITY trial also supports the benefit of achieving histologic improvement [42]. Narula et al. demonstrated that a change in epithelial neutrophilic infiltrate (the defining feature of Geboes Grade 3) during induction was an accurate predictor of response (achievement of endoscopic and histologic improvement) to biologic therapy during maintenance treatment [42].

Results reported in this study also support previous evidence showing improved outcomes with an even stricter definition of mucosal healing. The HEMR endpoint (deep mucosal healing) combines both endoscopic and histologic remission cutoffs (MES = 0 and Geboes histologic score < 2.0, respectively) and requires that the mucosa appear normal upon endoscopic inspection, and that no acute inflammatory infiltrate (i.e., neutrophils and/or eosinophils) be present in either the crypts or lamina propria [31, 41]. The stricter definition of HEMR was associated with more favorable and durable long-term patient outcomes in our analysis. These results are consistent with previously published literature [12, 13, 42]. A longitudinal cohort study demonstrated that patients who achieved an MES of 0 were less likely to experience a relapse at 6 months than those with an MES of 1[12]. A meta-analysis confirmed that patients who achieved both of the more rigorous goals of endoscopic remission and histologic remission had a lower risk of clinical relapse at 1 year than patients who achieved only endoscopic remission [13]. Histologic remission (Geboes histologic score < 2.0) and its relation to patient outcomes, independent of endoscopic findings, was recently reviewed by Chateau et al., [14] and it was shown to be associated with more favorable prognoses and outcomes. The importance of early histologic remission is suggested by the findings of Choi et al. [43] who reported that the risk of colorectal neoplasia in UC is significantly correlated with the total amount of inflammatory damage accumulated over time. Histologic remission has also been associated with lower rates of high-grade dysplasia and colon cancer [44]. We also demonstrated that achievement of histologic remission (Geboes histologic < 2.0) at the end of induction or maintenance with upadacitinib was associated with the greatest likelihood of achieving desirable clinical and patient-reported outcomes after 1 year of maintenance treatment. Finally, our results show that HEMR has greater predictive power for clinical remission and endoscopic remission, as well as UC-SQ, compared to HEMI without HEMR, which further demonstrates the greater clinical relevance of this more stringent measure of deep mucosal healing. To our knowledge, no other clinical trials aside from U-ACHIEVE have evaluated HEMR as a primary or ranked secondary endpoint. Thus, the effects of other treatments on HEMR have not been evaluated. Until such studies are performed, it is difficult to predict whether findings obtained with other treatments would be similar to those obtained with upadacitinib.

Strengths of this study include patients’ receiving standard treatment and follow-up, as well as centralized histologic assessments as defined in the clinical trial protocol. Although previous studies have shown improved clinical outcomes with mucosal healing, these studies have not examined long-term outcomes in terms of histologic remission. Our study evaluated a unique composite endpoint of endoscopic histologic remission, which was defined as Mayo endoscopic score = 0 and Geboes histologic score < 2.0. To our knowledge, this is the first study that assesses the benefits of achieving mucosal healing based on this stringent endoscopic and histologic measure. We acknowledge a number of limitations in our study. One limitation of this study is that logistic regression analyses were not performed on hospitalizations and surgeries because of the limited number of patients who were hospitalized or had surgery in the upadacitinib data. While this study used data from a randomized clinical trial, residual confounding may still arise from factors not included as covariates in the logistic regression. In addition, although full colonoscopy was performed for all participants at screening, the biopsy specimen was taken only from the rectosigmoid region and it is possible that a more proximal segment may, in some cases, have a greater histologic disease burden than the rectosigmoid regions. Furthermore, our results are based on data from Phase 3 clinical trials, which may not correspond to outcomes observed in routine clinical practice. Finally, outcomes in the maintenance study were assessed after a relatively short period of time (at Week 52). However, additional data is currently being collected in an ongoing long-term extension study, which will allow analysis of outcomes for a longer period of time.

## Conclusions

Based on our analysis of upadacitinib maintenance trial data, the stringent mucosal healing endpoints, HEMI, and HEMR were both clinically relevant with HEMR being associated with the greatest likelihood of improvement in long-term clinical and patient-reported outcomes. Early HEMI and HEMR are independent predictors of later remission and improved quality of life. Data on achieving the endoscopic remission and histologic remission endpoints and their benefit on long-term outcomes in upadacitinib-treated patients support HEMI and HEMR as desirable treatment goals in UC.

### Supplementary Information

Below is the link to the electronic supplementary material.

Supplementary file1 (PDF 633 KB)

## Declarations

### Writing assistance

Medical writing assistance was provided by Joann Hettasch, Ph.D., of Fishawack Facilitate Ltd., part of Fishawack Health, and was funded by AbbVie Inc., North Chicago, IL.
