# Supplementary material for: Correlation of mucosal healing endpoints with long-term clinical and patient-reported outcomes in ulcerative colitis
Source: J Gastroenterol. 2023 Jul 25;58(10):990–1002. doi: 10.1007/s00535-023-02013-7 (PMC10522527; doi:10.1007/s00535-023-02013-7)
Supplement: Supplementary file 1 — Supplementary file1 (PDF 633 KB) [file 535_2023_2013_MOESM1_ESM.pdf]

SUPPLEMENTAL MATERIALS

Correlation of Mucosal Healing Endpoints with Long-Term Clinical and Patient-Reported Outcomes in Ulcerative Colitis

Gareth Parkes[1], Ryan C. Ungaro[2], Silvio Danese[3], Maria T. Abreu[4], Ethan Arenson[5], Wen Zhou[6], Dapo Ilo[6], F. Stephen Laroux[7], Huiwen Deng[6,8], Yuri Sanchez Gonzalez[6], Laurent Peyrin-Biroulet[9]

[1]Barts Health NHS Trust, London, UK; Barts and the London School of Medicine and Dentistry, London, UK; [2]Division of Gastroenterology, Icahn School of Medicine at Mount Sinai, New York, NY, USA; [3]Gastroenterology and Endoscopy, IRCCS Ospedale San Raffaele, Milano Italy; [4]Division of Gastroenterology, Crohn's and Colitis Center, University of Miami Miller School of Medicine, Miami, FL, USA; [5]Adelphi Values, Boston, MA, USA; [6]AbbVie Inc., Chicago, IL, USA; [7]AbbVie Bioresearch Center Worcester, MA, USA; [8]Department of Pharmacy Systems Outcomes and Policy, University of Illinois at Chicago, Chicago, IL, USA; [9]University Hospital of Nancy, Lorraine University, Vandoeuvre, France

Corresponding author:
Gareth Parkes
Email: gareth.parkes@nhs.net

Journal of Gastroenterology

Supplemental Table 1: Cross-sectional analyses: adjusted odds ratios for HEMR or HEMI at Week 52 (end of maintenance) and clinical outcomes at Week 52 in U-ACHIEVE

Outcomes at Week 52[a]	Separate regressions for HEMR and HEMI			
	HEMR (n=55[b]) vs no HEMR (n=146[b])		HEMI (n=124[b]) vs no HEMI (n=77[b])	
	Adjusted Odds ratio (95% CI)[c]	*P* value	Adjusted Odds ratio (95% CI)[c]	*P* value
**Clinical Outcomes**				
Corticosteroid-free remission[b,d]	19.1 (6.6, 55.3)	<.001	179.8 (37.5, 861.7)	<.001
Sustained clinical response[b,e]	11.1 (2.6, 47.5)	.001	18.4 (5.8, 58.2)	<.001
Clinical remission per full Mayo score[b,f]	67.1 (16.3, 275.7)	<.001	128.5 (32.1, 513.9)	<.001
Clinical remission per adapted Mayo score[b,g]	31.3 (8.9, 109.9)	<.001	373.3 (66.1, >999.99)	<.001
Endoscopic remission[b,h]	N/A due to HEMR defintion		158.4 (17.3, >999.99)	<.001
Clinical and endoscopic remission per full Mayo score [b,f,h]	>999.99 (149.0, >999.99)	<.001	104.7 (12.3, 889.5)	<.001
Clinical and endoscopic remission per adapted Mayo score [b,g,h]	>999.99 (149.0, >999.99)	<.001	104.7 (12.3, 889.5)	<.001
Rectal bleeding = 0[b]	12.5 (2.3, 68.1)	.004	12.3 (3.7, 40.5)	<.001
Stool frequency ≤1[b]	17.1 (4.3, 68.0)	<.001	9.3 (4.0, 21.8)	<.001
Endoscopic improvement[b,i]	N/A due to HEMR definition		N/A due to HEMI definition	
FCP ranges[j]				
FCP ≤150 µg/g	3.5 (1.6, 7.6)	.002	4.9 (2.3, 10.5)	<.001
FCP ≤250 µg/g	3.7 (1.6, 8.5)	.003	3.9 (1.9, 8.2)	<.001
**Clinically meaningful improvement from induction baseline in patient-reported outcomes[k]**				
FACIT-F (≥5)[b]	1.6 (0.8, 3.4)	.182	1.7 (0.9, 3.3)	.124
UC-SQ (≥10)[b]	3.0 (1.2, 7.4)	.020	3.5 (1.7, 7.4)	<.001
IBDQ (≥16)[b]	2.3 (0.8, 6.1)	.111	5.3 (2.3, 12.6)	<.001
SF-36 PCS (≥4.1)[j]	2.5 (1.1, 5.8)	.038	3.4 (1.6, 7.3)	.002

Outcomes at Week 52[a]	Separate regressions for HEMR and HEMI			
	HEMR (n=55[b]) vs no HEMR (n=146[b])		HEMI (n=124[b]) vs no HEMI (n=77[b])	
	Adjusted Odds ratio (95% CI)[c]	*P* value	Adjusted Odds ratio (95% CI)[c]	*P* value
SF-36 MCS (≥4.1)[j]	3.8 (1.7, 8.5)	.001	2.8 (1.4, 5.7)	.006
EQ-5D-5L index (≥0.076)[j]	2.1 (0.9, 4.5)	.073	4.6 (2.1, 10.2)	<.001
WPAI				
Work time missed ≥6.5[l]	0.8 (0.3, 2.3)	.655	1.1 (0.4, 2.8)	.893
Impairment while working ≥6.1[l]	1.7 (0.3, 9.6)	.529	2.9 (0.7, 11.4)	.139
Overall work impairment ≥7.3[l]	2.3 (0.4, 12.0)	.335	3.1 (0.8, 11.8)	.100
Activity impairment ≥8.5	1.9 (0.7, 5.1)	.195	4.1 (1.7, 9.8)	.001

[a]NRI-NC was conducted in all Week 52 outcomes.
[b]N = 201, includes patients who achieved a clinical response after 8 weeks or 16 weeks of upadacitinib induction treatment.
[c]Adjusted for maintenance baseline Geboes histologic score, dosage, gender, age, weight, UC disease extent, UC disease duration, and use of extended therapy (16-week induction period). Due to the adjustment of covariates, patients with missing values on the covariates were dropped in the logistic regressions.
[d]Steroid-free remission was defined as achieved 90-day steroid-free remission per adapted Mayo in patients who achieved clinical remission at the end of induction treatment.
[e]Sustained clinical response was defined as remained clinically responsive at the end of Week 52.
[f]Clinical remission per full Mayo was defined as total Mayo score ≤2 with no subscore >1.
[g]Clinical remission per adapted Mayo (full Mayo excluding physician's global assessment) was defined as stool frequency subscore ≤1 and not greater than baseline, rectal bleeding subscore = 0, endoscopic subscore ≤1 without friability.
[h]Endoscopic remission defined as Mayo endoscopic subscore = 0.
[i]Endoscopic improvement defined as Mayo endoscopic subscore ≤1.
[j]N = 172, includes only patients who achieved a clinical response after 8 weeks of upadacitinib induction treatment.
[k]Clinically meaningful improvement in patient-reported outcomes was assessed as the likelihood of achieving a change from induction baseline in patient-reported outcome score ≥ the corresponding meaningful within-patient change threshold.
[l]Only includes patients who had baseline WPAI scores. N=154 for work time missed, N=142 for impairment while working, N=154 for overall work impairment.

CI = confidence interval; EQ-5D-5L, European Quality of Life Five Dimensions Five Levels; FACIT-F = Functional Assessment of Chronic Illness Therapy–Fatigue; FCP = fecal calprotectin; HEMI = histologic endoscopic mucosal improvement; HEMR = histologic endoscopic mucosal remission; IBDQ = Inflammatory Bowel Disease Questionnaire; N/A = not applicable; NRI-NC = non-responder imputation with no special data handling for missing due to COVID-19; SF-36 MCS = Short Form Health Survey Mental Component Summary; SF-36 PCS = Short Form Health Survey Physical Component Summary; UC = ulcerative colitis; UC-SQ = Ulcerative Colitis Symptoms Questionnaire: WPAI = Work Productivity and Activity Impairment Questionnaire.

Supplemental Table 2. Predictive analyses: adjusted odds ratios for HEMR or HEMI at Week 8/16 (end of induction) and outcomes at Week 52 (end of maintenance) in U-ACHIEVE

Outcomes at Week 52[a]	Separate regressions for HEMR and HEMI			
	HEMR (n = 45[b]) vs no HEMR (n = 275[b])		HEMI (n = 123[b]) vs no HEMI n = 197[b])	
	Adjusted Odds ratio (95% CI)[c]	*P* value	Adjusted Odds ratio (95% CI)[c]	*P* value
**Clinical Outcomes**				
Corticosteroid-free remission[b,d]	2.6 (1.3, 5.4)	.009	3.1 (1.8, 5.3)	<.001
Sustained clinical response[b,e]	2.3 (1.0, 5.1)	.042	2.1 (1.2, 3.7)	.006
Clinical remission per full Mayo score[b,f]	1.7 (0.8, 3.4)	.157	2.8 (1.6, 4.7)	<.001
Clinical remission per adapted Mayo score[b,g]	2.4 (1.2, 4.9)	.018	3.3 (1.9, 5.6)	<.001
Endoscopic remission[b,h]	2.7 (1.3, 5.8)	.008	2.8 (1.6, 5.2)	<.001
Clinical and endoscopic remission per full Mayo score [b,g,h]	2.1 (1.0, 4.6)	.051	2.8 (1.5, 5.2)	<.001
Clinical and endoscopic remission per adapted Mayo score [b,g,h]	2.3 (1.1, 5.1)	.029	2.9 (1.6, 5.4)	<.001
Rectal bleeding = 0[b]	2.8 (1.2, 6.8)	.018	2.2 (1.2, 3.8)	.006
Stool frequency ≤1[b]	2.1 (1.0, 4.6)	.054	2.2 (1.3, 3.7)	.003
Endoscopic improvement[b,i]	3.3 (1.5, 7.1)	.002	3.7 (2.1, 6.4)	<.001
FCP ranges[j]				
FCP ≤150 µg/g	2.0 (1.0, 4.1)	.073	1.8 (1.1, 3.1)	.027
FCP ≤250 µg/g	2.6 (1.2, 5.5)	.016	2.3 (1.3, 4.0)	.003
**Clinically meaningful improvement from induction baseline in patient-reported outcomes[k]**				
FACIT-F (≥5)[b]	1.4 (0.7, 2.8)	.340	1.2 (0.7, 2.0)	.462
UC-SQ (≥10)[b]	2.4 (1.1, 5.2)	.031	1.7 (1.0, 2.8)	.051
IBDQ (≥16)[b]	1.3 (0.6, 2.8)	.496	1.1 (0.7, 2.0)	.634
SF-36 PCS (≥4.1)[j]	1.1 (0.5, 2.3)	.816	1.3 (0.8, 2.3)	.287
SF-36 MCS (≥4.1)[j]	1.3 (0.6, 2.6)	.508	1.0 (0.6, 1.8)	.888
EQ-5D-5L index (≥0.076)[j]	1.5 (0.7, 3.2)	.267	0.9 (0.5, 1.6)	.768

Outcomes at Week 52[a]	Separate regressions for HEMR and HEMI			
	HEMR (n = 45[b]) vs no HEMR (n = 275[b])		HEMI (n = 123[b]) vs no HEMI n = 197[b])	
	**Adjusted Odds ratio (95% CI)[c]**	***P* value**	**Adjusted Odds ratio (95% CI)[c]**	***P* value**
WPAI				
Work time missed ≥6.5[l]	0.9 (0.3, 3.1)	.924	0.7 (0.3, 1.6)	.428
Impairment while working ≥6.1[l]	1.3 (0.3, 6.2)	.707	2.4 (0.8, 7.2)	.106
Overall work impairment ≥7.3[l]	1.4 (0.3, 6.5)	.648	2.0 (0.7, 5.7)	.174
Activity impairment ≥8.5	1.2 (0.5, 2.7)	.672	2.1 (1.1, 3.8)	.019

[a]NRI-NC was conducted in all Week 52 outcomes. No missing data imputation was used for HEMI and HEMR at Week 8/16.
[b]N = 320, includes patients who achieved a clinical response after 8 weeks or 16 weeks of upadacitinib induction treatment.
[c]Adjusted for maintenance baseline Geboes histologic score, dosage, gender, age, weight, UC disease extent, UC disease duration, and use of extended therapy (16-week induction period). Due to the adjustment of covariates, patients with missing values on the covariates were dropped in the logistic regressions.
[d]Steroid-free remission was defined as achieved 90-day steroid-free remission per adapted Mayo in patients who achieved clinical remission at the end of induction treatment.
[e]Sustained clinical response was defined as remained clinically responsive at the end of Week 52.
[f]Clinical remission per full Mayo was defined as total Mayo score ≤2 with no subscore >1.
[g]Clinical remission per adapted Mayo (full Mayo excluding physician's global assessment) was defined as stool frequency subscore ≤1 and not greater than baseline, rectal bleeding subscore = 0, endoscopic subscore ≤1 without friability.
[h]Endoscopic remission defined as Mayo endoscopic subscore = 0.
[i]Endoscopic improvement defined as Mayo endoscopic subscore ≤1.
[j]N = 275, includes only patients who achieved a clinical response after 8 weeks of upadacitinib induction treatment.
[k]Clinically meaningful improvement in patient-reported outcomes was assessed as the likelihood of achieving a change from induction baseline in patient-reported outcome score ≥ the corresponding meaningful within-patient change threshold.
[l]Only includes patients who had baseline WPAI scores. N=154 for work time missed, N=142 for impairment while working, N=154 for overall work impairment.

CI = confidence interval; EQ-5D-5L, European Quality of Life Five Dimensions Five Levels; FACIT-F = Functional Assessment of Chronic Illness Therapy–Fatigue; FCP = fecal calprotectin; HEMI = histologic endoscopic mucosal improvement; HEMR = histologic endoscopic mucosal remission; IBDQ = Inflammatory Bowel Disease Questionnaire; NRI-NC = non-responder imputation with no special data handling for missing due to COVID-19; SF-36 MCS = Short Form Health Survey Mental Component Summary; SF-36 PCS = Short Form Health Survey Physical Component Summary; UC = ulcerative colitis; UC-SQ = Ulcerative Colitis Symptoms Questionnaire; WPAI = Work Productivity and Activity Impairment questionnaire.

Supplemental Table 3. Spearman correlations between Geboes score and secondary instruments at Baseline and Week 8.

Secondary measures	N=491	Baseline	N=491	Week 8
Mayo: stool frequency subscore	490	0.17	443	0.43
Mayo: rectal bleeding frequency subscore	490	0.08	443	0.37
Mayo: endoscopy subscore	491	0.10	427	0.53
Mayo: Physician's global assessment	491	0.13	444	0.41
Mayo Full score	490	0.20	424	0.54
Mayo Partial score	490	0.19	443	0.49

Supplemental Table 4. Predictive analysis: patients with ≥ 1 UC-related hospitalization or surgery through the maintenance phase in U-ACHIEVE stratified by mutually exclusive HEMI and HEMR categories at Week 8/16 (end of induction)

At Week 8/16	N	≥ 1 UC-related hospitalization or surgery	UC-related hospitalizations	UC-related surgeries
No HEMI	197	3	2	1
HEMI without HEMR	78	1	1	0
HEMR	45	0	0	0

HEMI = histologic endoscopic mucosal improvement; HEMR = histologic endoscopic mucosal remission; N = number of patients; UC = ulcerative colitis.

Supplemental Table 5. Cross-sectional analysis: Patients with ≥ 1 UC-related hospitalization or surgery through the maintenance phase in U-ACHIEVE stratified by mutually exclusive HEMI and HEMR categories at Week 52

At Week 52	N	≥ 1 UC-related hospitalization or surgery	UC-related hospitalizations	UC-related surgeries
No HEMI	77	2	1	1
HEMI without HEMR	69	0	0	0
HEMR	55	0	0	0

HEMI = histologic endoscopic mucosal improvement; HEMR = histologic endoscopic mucosal remission; N = number of patients; UC = ulcerative colitis.

Correlation of histologic assessment of mucosal healing with long-term clinical outcomes in patients participating in the U-ACHIEVE maintenance trial

METHODS

Data Source

Geboes score (GS) and long-term clinical outcomes from the upadacitinib Phase 3 U-ACHIEVE maintenance trial (NCT02819635) were analyzed. GS intervals were selected to assess: (1) patients who achieved the threshold for histologic remission (GS <2.0); (2) patients who achieved the threshold for histologic improvement without remission (2.0 ≤ GS ≤3.1) (3) patients who did not achieve histologic improvement (GS >3.1). Outcomes were examined in patients who achieved a clinical response after 8 or 16 weeks of upadacitinib induction treatment and received 15 mg or 30 mg QD dose of upadacitinib during maintenance.

Data Analysis

A cross-sectional analysis (N = 211) examined the relationship of both GS intervals and outcomes at the end of maintenance (Week 52). A predictive analysis (N = 497) assessed the relationship between GS intervals at the end of induction (Week 8/16) and outcomes at the end of maintenance (Week 52). Odds ratios (ORs) with 95% confidence intervals (CIs) were derived from logistic regression adjusting for the following characteristics: histologic score at baseline, maintenance treatment dosage, gender, disease extent, disease duration, and baseline age and weight.

Results

Patients with histologic remission (GS <2.0) at Week 52 had a significantly greater likelihood of achieving all clinical outcomes at Week 52 compared to patients with GS >3.1 (Supplemental Figure 1). Patients with histologic remission (GS <2.0) at end of induction (Week 8/16) had a significantly higher likelihood of achieving clinical remission per adapted Mayo score (OR = 1.8, P <.030) and endoscopic improvement (OR = 1.8, P <.015) at Week 52 than patients with GS >3.1 (Supplemental Figure 2).

Supplemental Figure 1. Cross-sectional analysis Adjusted ORs of Clinical Outcomes at Week 52 for GS categories at Week 52 in U-ACHIEVE

Maintenance Phase

Clinical Outcomes	OR (95% CI)
Clinical remission per full Mayo[a]	14.9 (4.7, 46.8)***
	6.6 (1.6, 27.2)**
Clinical remission per adapted Mayo[a]	20.4 (6.3, 65.5)***
	13.0 (3.2, 53.2)***
Endoscopic remission[a]	32.8 (3.9, 273.3)**
	16.3 (1.6, 166.4)*
Clinical and endoscopic remission per full Mayo[a]	25.5 (3.2, 203.7)**
	10.9 (1.1, 110.3)*
Clinical and endoscopic remission per adapted Mayo[a]	25.5 (3.2, 203.7)**
	10.9 (1.1, 110.3)*
Rectal bleeding = 0[a]	6.5 (2.4, 17.4)***
	4.0 (0.9, 17.0)
Stool frequency ≤1[a]	11.7 (4.7, 29.2)***
	7.2 (2.0, 25.8)**
Endoscopic improvement[a]	26.7 (9.0, 79.1)***
	11.7 (3.0, 45.5)***
FCP ≤150[b]	4.5 (1.6, 12.5)**
	2.2 (0.6, 8.6)
FCP ≤250[b]	5.8 (2.1, 16.0)***
	2.8 (0.7, 10.7)

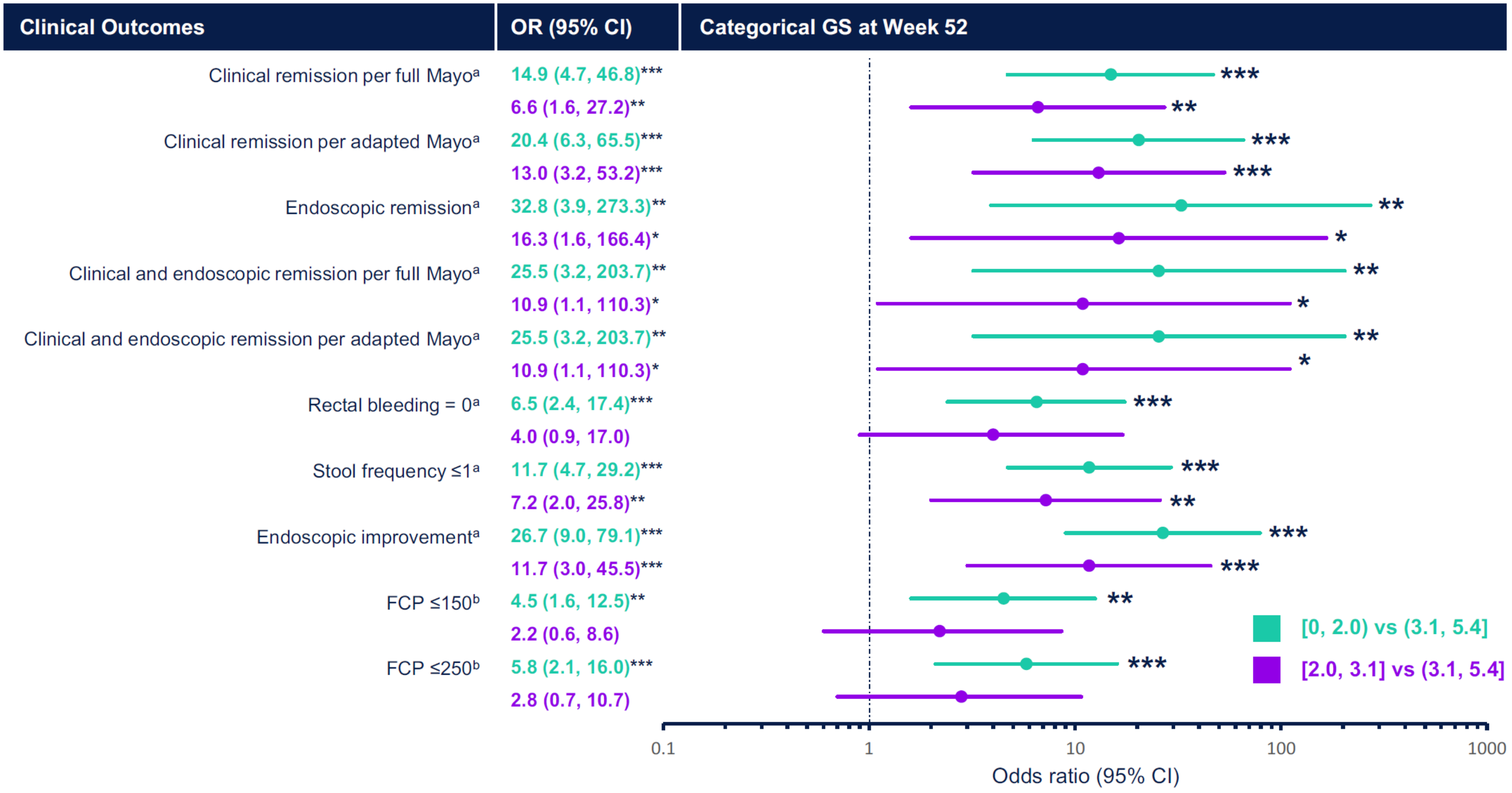

*P <.05, **P <.01, ***P <.001. An OR >1 indicates an increased likelihood, whereas an OR <1 indicates a decreased likelihood of achieving the outcome.

[a]N = 211, includes patients who achieved a clinical response after 8 weeks or 16 weeks of upadacitinib induction treatment.

[b]N = 182, includes only patients who achieved a clinical response after 8 weeks of upadacitinib induction treatment.

Supplemental Figure 2. Predictive Analysis: Adjusted ORs of Clinical Outcomes at Week 52 for GS Categories at Week 8/16 in U-ACHIEVE

Induction Phase

Clinical Outcomes	OR (95% CI)
Clinical remission per full Mayo[a]	1.6 (1.0, 2.7)
	1.1 (0.6, 2.0)
Clinical remission per adapted Mayo[a]	1.8 (1.1, 3.0)*
	1.1 (0.6, 2.0)
Endoscopic remission[a]	1.8 (0.9, 3.6)
	1.1 (0.5, 2.4)
Clinical and endoscopic remission per full Mayo[a]	1.9 (0.9, 3.8)
	1.0 (0.4, 2.3)
Clinical and endoscopic remission per adapted Mayo[a]	2.0 (1.0, 4.2)
	1.1 (0.4, 2.6)
Rectal bleeding = 0[a]	1.1 (0.7, 1.8)
	1.0 (0.6, 1.6)
Stool frequency ≤1[a]	1.2 (0.8, 1.9)
	0.9 (0.5, 1.5)
Endoscopic improvement[a]	1.8 (1.1, 3.0)*
	1.2 (0.7, 2.1)
FCP ≤150[b]	1.2 (0.7, 2.1)
	0.6 (0.3, 1.1)
FCP ≤250[b]	1.2 (0.7, 2.1)
	0.6 (0.3, 1.2)

Categorical GS at Week 8/16

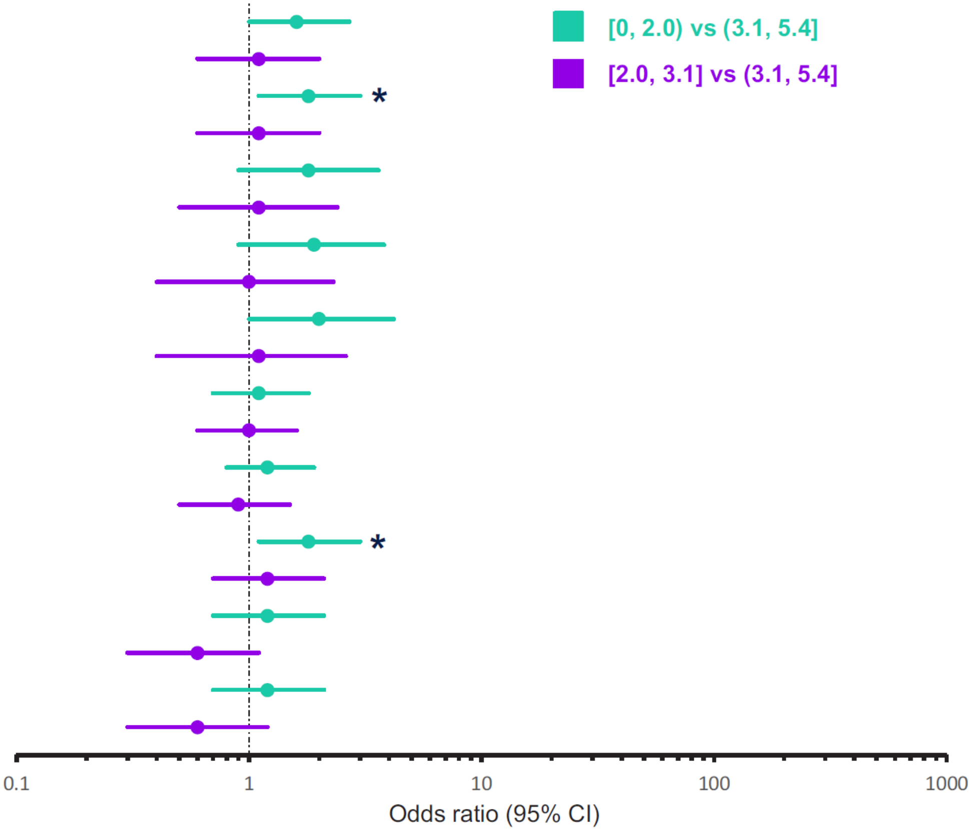

*P <.05. An OR >1 indicates an increased likelihood, whereas an OR <1 indicates a decreased likelihood of achieving the outcome.

[a]N = 497, includes patients who achieved a clinical response after 8 or 16 weeks of upadacitinib induction treatment.

[b]N = 427, includes only patients who achieved a clinical response after 8 weeks of upadacitinib induction treatment.
